# Supplementary material for: A Global Screen for Assembly State Changes of the Mitotic Proteome by SEC-SWATH-MS
Source: Cell Syst. 2020 Feb 26;10(2):133–155.e6. doi: 10.1016/j.cels.2020.01.001 (PMC7042714; doi:10.1016/j.cels.2020.01.001)

P25098 | ARBK1_HUMAN | GRK2 ADRBK1 BARK BARK1
Monomer MW [kDa]: 79.574 Monomer expected elution fraction: 42

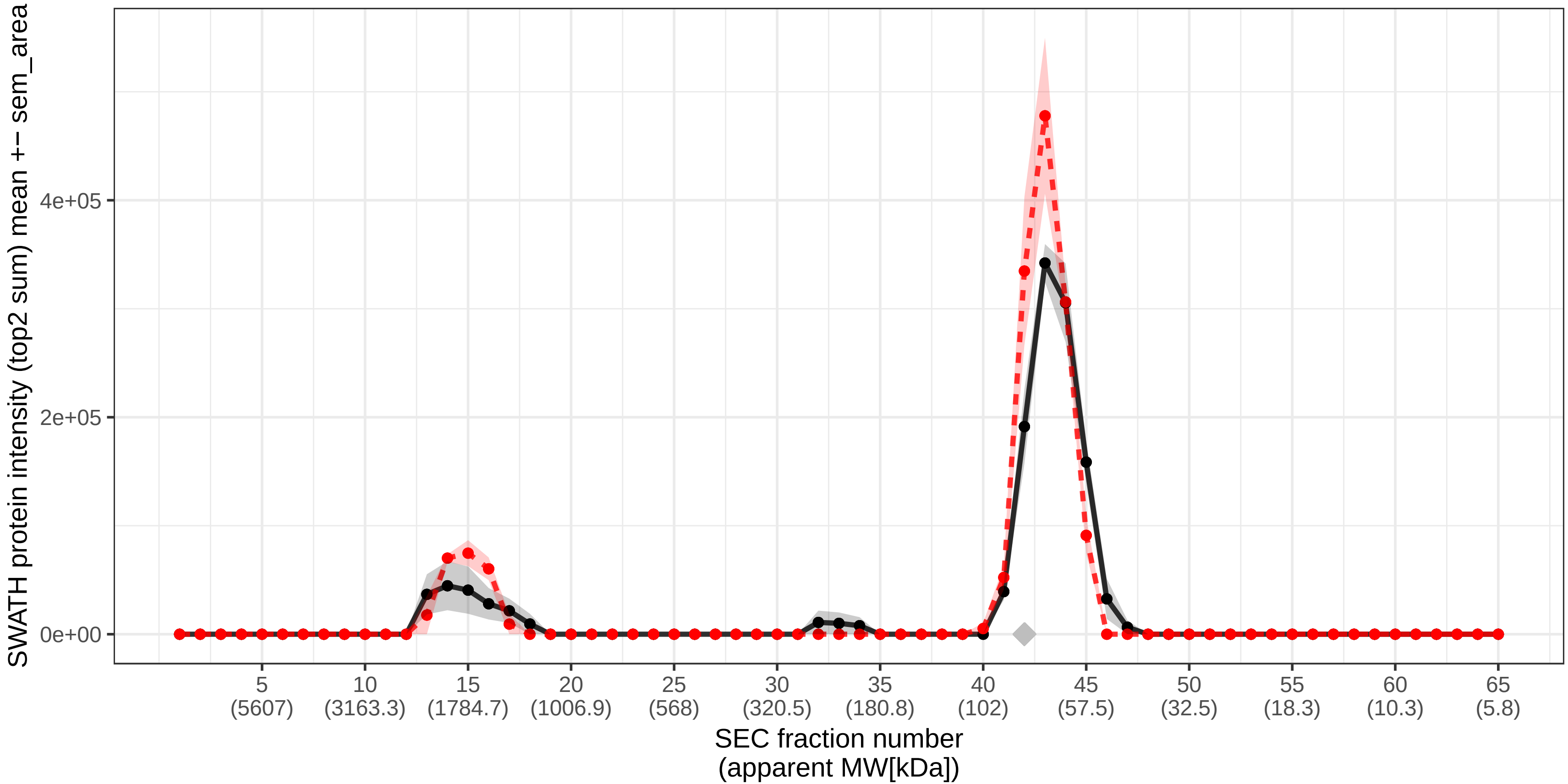

Supplement: Data S1. SEC-SWATH-MS Protein Chromatograms, Related to Figure 1 [file mmc6.zip › SECchrom_P25098_ARBK1_HUMAN_GRK2_ADRBK1_BARK_BARK1.pdf]
